# Comparison of Clinical Characteristics Between Hereditary Angioedema Patients Aged 65 Years and Older and Those Under 65: A Perspective on Elderly Patients

**DOI:** 10.3390/life16010122

**Published:** 2026-01-14

**Authors:** Gülseren Tuncay, Ebru Damadoglu, Gül Karakaya, Ali Fuat Kalyoncu

**Affiliations:** 1 Institute of Allergology, Charité—Universitätsmedizin Berlin, 13353 Berlin, Germany; 2 Fraunhofer Institute for Translational Medicine and Pharmacology ITMP, Immunology and Allergology, 12203 Berlin, Germany; 3 Department of Internal Medicine, Immunology and Allergy, Adiyaman Training and Research Hospital, 02040 Adiyaman, Turkey; 4 Division of Allergy and Clinical Immunology, Faculty of Medicine, Hacettepe University, 06230 Ankara, Turkey

**Keywords:** hereditary angioedema, angioedema control, C1INH, short-term prophylaxis, HAE-C1INH, elderly

## Abstract

**Background:** This study aimed to comprehensively define the clinical profile of elderly patients with hereditary angioedema (HAE) caused by C1 esterase inhibitor (C1INH) deficiency and/or dysfunction (HAE-C1INH). Furthermore, it sought to reveal age-related differences in disease expression and management by comparing these patients with their younger counterparts. **Methods:** In this retrospective study, seventy-six patients were included. All patients had been diagnosed with HAE-C1INH. **Results:** A total of 9 (12%) patients were ≥65 years, 7 (77%) of whom were female. The median age at the time of diagnosis was higher in the elderly group, whereas the median age at the first symptom was similar. There was a significant delay in diagnosis time in the elderly group. Hypertension was the most frequent comorbidity among elderly patients. The median number of angioedema attacks in the last year was 6, and similar to 10 in patients < 65 years. Angioedema control in the last three months was lower in older patients. The rate of laryngeal edema was similar in patients < 65 years and older patients. The use of short-term prophylaxis (STP) was higher in the elderly group. The most commonly used treatment for acute attacks was pdC1-INH. Two patients in the elderly group did not benefit from danazol. No adverse events with icatibant, pdC1-INH, danazol were encountered among patients. **Conclusions:** Compared to patients younger than 65 years of age, annual attack rates were similar, whereas elderly patients had lower angioedema control for the last three months. The use of STP rates was higher among elderly patients.

## 1. Introduction

Hereditary angioedema (HAE) due to C1 inhibitor (C1INH) deficiency and/or dysfunction (HAE-C1INH) is a rare, potentially life-threatening disorder characterized by recurrent episodes of subcutaneous and submucosal edema [1,2]. The prevalence of HAE due to C1 inhibitor deficiency is estimated to be approximately 1 in 50,000 individuals worldwide, and accounts for about more than 95% of all HAE cases [1,3,4]. These attacks often occur spontaneously and may involve the extremities, gastrointestinal tract, or upper airways, the latter posing a substantial risk of asphyxiation [3]. Although the clinical presentation and management of HAE-C1INH have been well documented in the general population, data focusing specifically on elderly patients remain limited. Advances in diagnostic methods and therapeutic options have allowed more patients to reach older ages, raising new questions regarding the influence of aging, comorbidities, and polypharmacy on disease course and treatment response [4,5]. Despite these developments, most available evidence is derived from younger cohorts, leaving an important gap in understanding the unique clinical features and management needs of older adults with HAE-C1INH. Identifying age-related differences may guide more individualized therapeutic approaches and improve outcomes in this potentially vulnerable subgroup. Therefore, the present study aimed to characterize the clinical profile of elderly patients with HAE-C1INH and compare them with younger patients followed at the same tertiary care center.

## 2. Materials and Methods

Descriptive analyses were conducted in younger patients (defined as ages < 65 years) versus elderly patients (≥65 years). Patients with a diagnosis of HAE-C1INH in Division of Immunology and Allergy, Hacettepe University Faculty of Medicine between 1 January 2001, and 31 December 2023, were retrospectively included in the study. The diagnosis of HAE-C1INH was established based on C1 inhibitor levels and function, as well as C4 levels. SERPING1 gene mutation analysis or next-generation sequencing was not performed in the patients. Only patients aged 18 years and above were screened, and those with normal C1INH levels were excluded. Written informed consent was obtained from all patients prior to participation in the study. Information regarding year of diagnosis, attack localizations, number and duration of attacks, triggering factors (minor trauma [e.g., impact, pressure, surgical procedures], dental procedures and other invasive medical interventions, prolonged standing or intense physical exertion, stress, emotional tension and anxiety, upper respiratory tract infections, febrile illnesses, estrogen-containing medications [oral contraceptives, hormone replacement therapy], menstrual cycle, pregnancy), medications used during attacks, the number of life-threatening episodes, treatments used for acute attacks, short-term prophylaxis (STP) or long-term prophylaxis (LTP), attack frequency and involvement sites under treatment, and the presence of attacks before or after surgical or interventional procedures was obtained from patient files and the hospital database. A standardized “data collection form” was created to ensure completeness of clinical data.

### Management of HAE-C1INH Treatments in the Study Population

The treatment of HAE has traditionally been divided into three groups: acute treatment, STP, and LTP [6]. In our country, plasma-derived C1 esterase inhibitors (pdC1-INH) are used as the first-line treatment and can be administered for acute attack management, STP prior to surgical procedures, and LTP [7]. For LTP, a dose of 1000–2500 IU twice weekly is recommended, although in practice 1000 IU is generally administered twice weekly or every 3–4 days. In addition to intravenous pdC1-INH, icatibant is also used for the treatment of HAE attacks [7]. All icatibant products available in our country are supplied as prefilled syringes containing 30 mg/3 mL solution, and they have been available for prescription since 2019. A maximum of three doses may be administered within 24 h, with at least a 6 h interval between doses [8]. When first-line therapies are not accessible, guidelines indicate that fresh frozen plasma (FFP) may be used as a second-line treatment for acute attacks [9]. An initial dose of two units of plasma is administered, and this dose may be repeated every 2–4 h until clinical improvement is achieved [7]. Attenuated androgens such as danazol are also considered safe alternatives to C1-INH concentrate for STP, although pdC1-INH remains the first-line option [9]. For procedures planned in advance, danazol prophylaxis is initiated five days prior to the intervention and continued until the second or third day after the procedure. Its oral route of administration facilitates ease of use. The optimal daily dose of danazol for the treatment of LTP to minimize adverse effects is ≤200 mg. However, initial doses of up to 400 mg per day may be used and subsequently reduced by approximately 50%, depending on the patient’s clinical response [7,10,11].

Statistical analyses were performed using SPSS version 25.0. Descriptive data were presented as numbers (n) and percentages (%). Numerical variables with normal distribution were expressed as mean ± standard deviation, whereas non-normally distributed variables were expressed as median and interquartile range. Categorical variables were compared using the Chi-square or Fisher’s exact test. Continuous variables were analyzed using the Mann–Whitney U test. Correlation analyses were performed using Spearman’s correlation coefficient for non-normally distributed variables. A *p*-value < 0.05 was considered statistically significant.

## 3. Results

As of 31 December 2023, a total of 76 patients with HAE-C1INH were included in the study, of whom 44 (58%) were female. The median age was 44 years (IQR: 35–58). Sixty-seven (88%) patients were younger than 65 years, while nine (12%) were ≥65 years, of whom seven (78%) were female. The median age at diagnosis was significantly higher in the elderly group compared with the younger group (58 vs. 28 years), whereas the age at first symptom onset was similar in both groups and was around 20 years old. The time interval from first symptoms to diagnosis was markedly longer in elderly patients (42 vs. 4 years). A family history of HAE was present in 44% of elderly patients and 75% of younger patients (*p* = 0.06). Among patients with a family history, diagnostic delay was markedly longer in the elderly group compared with younger counterparts (46.5 vs. 25.9 years, *p* = 0.008). However, when there was no family history, the delay was comparable between the two age groups (*p* = 0.15). In the group aged <65 years, the most common comorbidity was thyroid disease (37%), while hypertension (80%) was the predominant comorbidity in the elderly group. The clinical characteristics of the two age groups are summarized in Table 1.

The median annual number of angioedema (AE) attacks was 6 in the elderly group and 10 in the younger group. Nonetheless, the frequency of emergency department visits due to AE attacks was similar across groups. The anatomical distribution of AE in the older group was also comparable, most frequently involving the face (78%), abdomen (78%), the extremities (44%), and throat (33%), respectively.

**Figure 1:**
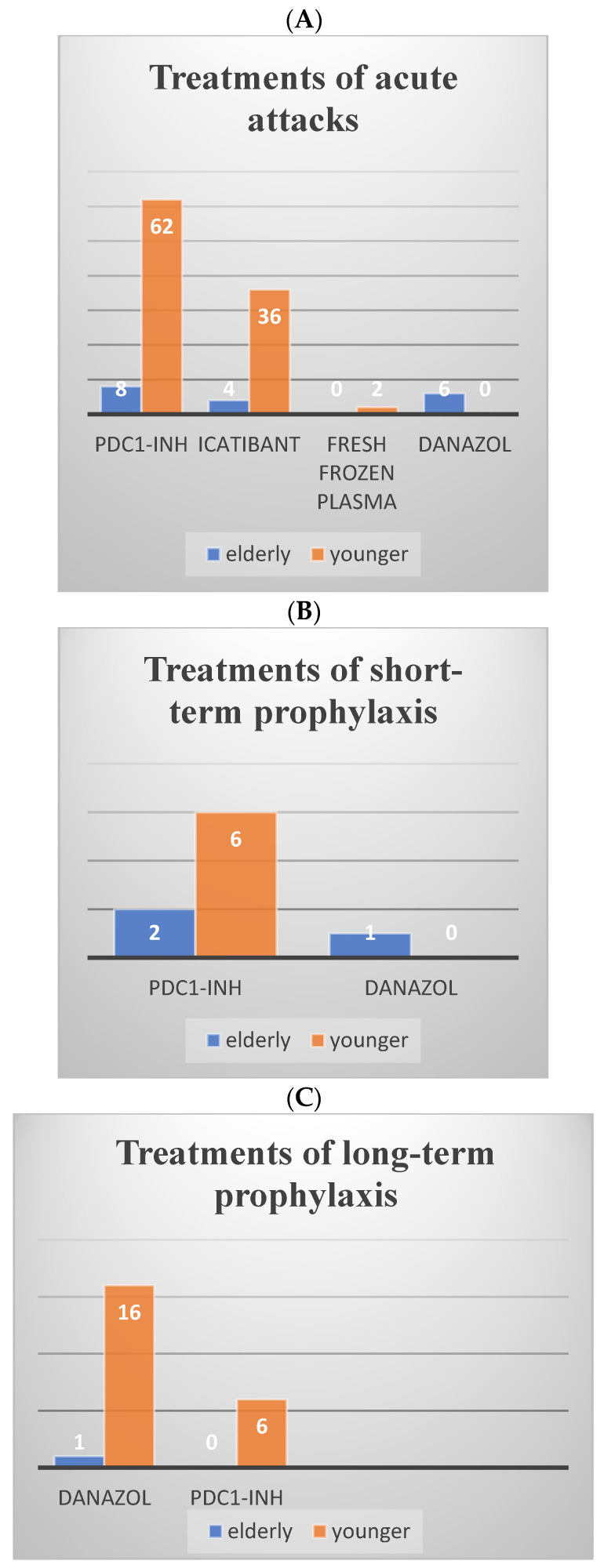
Elderly (n = 9) and younger (n = 67) patients taking on-demand (**A**), short-term (**B**) and long-term (**C**) prophylaxis therapy at the time of data recording. pdC1-INH: I.V.

AECT-3 month scores did not differ significantly between the groups; however, the proportion of patients with uncontrolled disease was higher among the elderly. For acute attacks, pdC1-INH was the most frequently administered treatment in both groups, followed by icatibant. FFP was used only in two (3%) younger patients. STP was more commonly used in the elderly group, and danazol was administered to one (11%) elderly patient. For LTP, danazol was the most frequently used agent in both groups, with comparable rates of use. Treatments were summarized in Figure 1.

Danazol therapy was discontinued in six patients overall (two in the elderly group) due to inadequate disease control. Among patients younger than 65 years, danazol was additionally discontinued in three individuals (4%) due to adverse effects, including weight gain, excessive hair growth, or menstrual irregularities. No cases of icatibant non-responsiveness were identified in the elderly group.

## 4. Discussion

Patients aged ≥65 years diagnosed with HAE-C1INH constituted 12% of the study population and were predominantly female (78%). The age at which the first symptoms appeared was similar in both groups. The time from the onset of the first symptoms to diagnosis was significantly longer in elderly patients compared to younger patients. Hypertension was the most common comorbidity in the elderly (80%), whereas thyroid disease predominated in younger patients (37%). The family history of HAE and the annual AE attack rates, and AE localizations were similar in both groups. Interestingly, among patients with a family history, the diagnostic delay was significantly longer in the elderly group compared with younger individuals (46.5 vs. 25.9 years). The proportion of patients whose disease could not be controlled was higher among the elderly. Whereas pdC1-INH was the most frequently administered agent for acute attacks and STP, danazol was the most commonly used therapy for LTP. In the elderly group, AE attacks could not be controlled with danazol and were discontinued in two patients.

In the literature, the prevalence of patients with HAE-C1INH aged 60 years and older ranges between 11% and 18%; similarly, this proportion was 12% in our study, consistent with previously reported data [12,13]. In our study, the diagnosis of HAE-C1INH was based on C1 inhibitor levels and function, as well as C4 levels. However, next-generation sequencing (NGS) analysis, one of the most widely used genetic diagnostic methods, was not performed. Consequently, potential diagnostic mutations and their correlation or discrepancies with clinical findings between elderly and younger patients could not be evaluated. A previous study has emphasized that NGS can identify novel mutations and may serve as a first-line approach for diagnostic confirmation, highlighting an area for future research [14].

The gender distribution was consistent with the literature, showing a female dominance, and was similar in both groups [15]. In a previous study evaluating 871 HAE patients and comparing those aged 65 and older with younger patients, the age at onset of symptoms was found to be similar in both groups at 17 years, but the median age in the older group in our cohort was 20 years. Additionally, while the median delay in diagnosis in the elderly group in our cohort was 42 years, it was 24 years in the study by Bygum and colleagues. It has been reported that the average delay in diagnosis in adults is 8–20 years, but different studies have emphasized that the diagnosis of the disease may stem from both the level of awareness among physicians and the fact that it cannot be detected in childhood [16,17,18,19,20]. In addition, delays in accessing physicians in different populations’ healthcare systems, the absence of severe angioedema attacks in some patients, or the lack of relatives with similar attacks who have been diagnosed with HAE-C1INH may also have contributed to these diagnostic delays. However, consistent with our study data, a family history of HAE was similarly observed in both groups [15]. Interestingly, in patients who had a positive family history, the elderly experienced a markedly longer diagnostic delay than the younger group. In contrast, when no family history was present, the delay did not differ substantially between the two age groups. This finding also supports the data reported in the study by Bygum and colleagues [15]. This could be explained by the markedly higher number of younger patients with a family history—allowing for earlier recognition—as well as by the improved diagnostic evaluation of affected families in contemporary clinical practice. In elderly patients with a family history, the prolonged delay may also reflect that their older relatives may have stopped seeking medical care and continued living with symptoms, as formal diagnosis and effective HAE-C1INH treatments were not available in earlier decades.

Compared with younger patients, elderly patients tended to have a higher prevalence of multiple comorbidities (56% vs. 28%, *p* = 0.10). This difference may stem from the heterogeneous distribution of the groups. However, in a previous study involving elderly HAE patients, hypertension was the most common comorbidity, consistent with our study [15]. A family history of HAE was observed to be more prevalent in the younger age group. In a study evaluating 872 patients with HAE-C1INH and comparing individuals aged 65 years and older with younger patients, the proportion of those with a family history was similar between the groups; however, this rate was 75% in the older cohort, whereas it was 44% in our study. The discrepancy between these rates may be attributable to asymptomatic family members who presented to reference centers but were not screened, insufficient screening of all family members after identification of the index case, varying levels of physician awareness, and challenges in differential diagnosis and in accessing HAE reference centers across different countries [16,21,22,23]. Consistent with the literature, the most common sites of AE involvement were the face and abdomen in the both groups [21]. In our study, in line with the existing literature, no difference was observed between the two groups regarding laryngeal involvement, and the larynx represented the fourth most common site of involvement [19]. In the literature, the rate of laryngeal edema has been reported to be approximately 1% to 5%; however, in our cohort, it was detected at rates exceeding 10% in both groups [19,24]. Nevertheless, uvula, tongue, and larynx were not classified as distinct sites of involvement, and the higher rate observed may have resulted from recall bias and/or the limited number of patients.

In the older group, 78% of the patients were female, and lack of disease control was more common in this group. Attack triggers in female patients are frequently described as menstruation, ovulation, pregnancy, and estrogen-containing hormones [25]. In a multicenter study involving 150 female patients with HAE-C1INH, 44 (29%) patients were postmenopausal. Among postmenopausal patients, 55% showed no change in attack frequency, 32% experienced worsening symptoms, and only 13% had less frequent symptoms [25]. In this study, we did not compare attack frequencies before and after menopause. However, lack of disease control was more common in the older group compared with younger patients. In addition, there was no difference in the frequency of comorbidities between the older and younger groups. In women with HAE, variations in sex hormone sensitivity have been described previously; for example, some women exhibit increased symptoms after puberty or during menstruation [26,27,28,29,30]. One possible explanation for these different phenotypes is the presence of mutations or polymorphisms in other potentially relevant genes, such as the angiotensin-converting enzyme gene, or the bradykinin B2 receptor gene [31]. Although data such as attack frequency, family history, and initial treatment responses in older patients—some of which may date back many years—were obtained from hospital records and patient self-reports, the potential factors such as recall bias and the limited availability of documentation related to past attacks should be taken into consideration.

Over the past 15 years, on-demand management has remained largely unchanged, with four approved targeted therapies available: intravenous plasma-derived or recombinant C1-INH, as well as subcutaneous options including the bradykinin B2 receptor antagonist icatibant and the kallikrein inhibitor ecallantide [9]. In our country, pdC1-INH and icatibant have been used for the treatment of acute AE attacks since August 2009 and January 2018, respectively. In a study evaluating the on-demand treatment preferences of 107 patients with HAE-C1INH, pdC1-INH was used in 1% of patients, whereas icatibant was used in 13%. However, only two patients (2%) aged ≥65 years were included in that study, and approximately 35% of the participants were receiving LTP with lanadelumab or berotralstat. In our cohort, by contrast, 12% of the patients were aged ≥65 years, and pdC1-INH was the most frequently used on-demand therapy (89%), while icatibant was used less frequently (44%). Current guidelines recommend STP with intravenous pdC1-INH, administered as close as possible to the procedure or triggering event, to lower the risk of post-procedure or stress-related HAE attacks [32]. However, in cases where first-line therapies are inaccessible or ineffective, danazol is also recommended for STP [33,34]. In our elderly cohort, pdC1-INH was used for STP in 67% of patients, whereas danazol was administered in 33% (as shown in Table 1). The use of STP was higher in this group compared with younger patients. This may be attributed to the higher frequency of medical, surgical, or dental interventions and other trigger factors for AE attacks in the elderly population [34,35].

In the study by Bork and colleagues, which evaluated 118 patients with HAE-C1INH receiving danazol as LTP, danazol was also used in patients aged ≥65 years, and an approximately 95% reduction in attack frequency was reported [36]. LTP use in our cohort was comparable between the two groups, and only one (11%) patient in the elderly group received danazol for LTP. Also, danazol was discontinued in two of the three elderly patients due to lack of response to treatment. More recently, a larger double-blind, randomized crossover trial confirmed these findings, with 111 of 118 patients showing a positive response to danazol. Additionally, 54 patients (46%) became symptom-free or experienced no more than one attack per year. Among the remaining patients, symptoms were generally mild, further supporting the effectiveness of danazol in preventing HAE attacks. However, danazol was discontinued in a total of 58 patients for various reasons; among these, lack of disease control was observed in seven (12%) patients. The oldest patient was 85 years old; however, the proportion of elderly patients among those who were non-responsive to danazol was not specified [36]. The limitations of androgen therapy in HAE have also been highlighted in several population-based studies. In a retrospective cross-sectional analysis of 650 individuals conducted in 2016, 186 patients discontinued androgen treatment, with lack of efficacy being one of the major reasons for discontinuation [37]. However, despite being a large population-based study, it did not include any patients aged 65 years or older. In a multicenter study evaluating the safety of icatibant in 872 patients (100 elderly and 772 younger), no serious adverse events were reported in the elderly group, and the incidence of mild and moderate adverse events was similar to that observed in younger patients [15]. Despite of previous studies, no cases of icatibant non-responsiveness were identified in our elderly group [32,38]. In the elderly HAE group in our cohort, the absence of adverse effects reported with any of the danazol or the icatibant, treatments suggest that these treatments are better tolerated in this group.

The retrospective design of the study, the small sample size of elderly patients, the limited treatment options, and the heterogeneity between groups constitute the main limitations of the study. In addition, the study was conducted at a single center, which limits the generalizability of the findings. However, as the first study comparing HAE-C1INH patients over and under 65 years of age, describing the clinical characteristics of elderly HAE-C1INH patients, and sharing treatment outcomes, the results obtained from this study provide a significant contribution to the literature. In future studies, multicenter designs and larger population data could enhance the generalizability of the findings.

## 5. Conclusions

While annual attack frequency and the rate of laryngeal involvement were similar between patients aged ≥65 years and those younger than 65, the elderly group experienced a significantly longer diagnostic delay and demonstrated poorer overall disease control. Despite these challenges, elderly patients showed good tolerability to icatibant, danazol, and pdC1-INH and exhibited higher rates of STP utilization. However, danazol seems to be less effective in achieving disease control in elderly patients, highlighting the need for further data on alternative treatment options, particularly for LTP. Moreover, as patients included in this study had limited access to modern long-term prophylactic agents such as lanadelumab and berotralstat, the generalizability of our findings should be confirmed by similar studies conducted in healthcare settings with broader access to these therapies. Overall, this descriptive analysis highlights both similarities and differences between HAE-C1INH patients aged 65 years and older and younger patients, emphasizing the importance of increased clinical awareness, timely diagnosis, and individualized management strategies to optimize disease control in older adults.

## Figures and Tables

**Table 1:** Demographic characteristics of the cohort: elderly versus younger subjects.

Characteristics	Patients < 65 Years Old (n = 67), (88%)	Patients ≥ 65 Years Old (n = 9), (12%)	*p*-Value
Age, median years (IQR)	42 (18–64)	69 (65–81)	**<0.001**
Female, n (%)	37 (55)	7 (78)	0.20
Age at the diagnosis, median years (IQR)	28 (5–58)	58 (49–69)	**<0.001**
Age at symptom onset, median years (IQR)	19 (3–50)	20 (7–58)	0.33
Delay in diagnosis, median years (IQR)	4 (1–50)	42 (1–50)	**0.002**
Duration of HAE ^1^, median years (IQR)	10 (1–37)	10 (1–20)	0.59
Comorbidities, n (%)	19 (28)	5 (56)	0.10
FMF ^2^	2 (10)	0 (0)	NA
Diabetes mellitus	3 (16)	2 (40)	NA
Hypertension	3 (16)	4 (80)	NA
Thyroid diseases	7 (37)	0 (0)	NA
Rhinitis	3 (16)	0 (0)	NA
Asthma	2 (10)	0 (0)	NA
Others *	6 (32)	1 (20)	NA
Family history with HAE, n (%)	50 (75)	4 (44)	0.06
The median number of attacks in the last year, (IQR)	10 (2–48)	6 (2–36)	0.82
Emergency admission due to angioedema attacks in the last year, n (%)	4 (4)	4 (10)	0.89
Localizations of the attacks, n (%)			
Face	58 (86)	7 (78)	0.72
Abdomen	42 (63)	7 (78)	0.37
Extremities	34 (51)	4 (44)	0.45
Larynx	10 (15)	3 (33)	0.17
AECT-3mth ^3^, median score (IQR)	13 (1–18)	10 (6–16)	0.13
<10 points, n (%)	11 (16)	4 (44)	**0.047**
Treatment of acute attacks, n (%)			
pdC1-INH ^4^	62 (92)	8 (89)	0.60
Icatibant	36 (54)	4 (44)	0.70
Fresh frozen plasma	2 (3)	0 (0)	NA
Short-term prophylaxis, n (%)	6 (9)	3 (33)	**0.04**
pdC1-INH	6 (100)	2 (67)	0.30
Danazol	0 (0)	1 (33)	NA
Long-term prophylaxis, n (%)	26 (39)	1 (11)	0.10
Danazol	16 (62)	1 (100)	0.38
pdC1-INH	6 (38)	0 (0)	NA
Unresponsiveness to treatment, n (%)	8 (44)	2 (22)	0.49
Icatibant	4 (50)	0 (0)	NA
Danazol	4 (50)	2 (100)	0.09
Treatments with AEs ^5^, n (%)	7 (12)	0 (0)	NA
Icatibant	4 (57)	0 (0)	NA
Danazol	3 (43)	0 (0)	NA

* Hyperlipidemia (n = 2), colon cancer (n = 2), gout (n = 1), chronic obstructive airway disease (n = 1), nephrolithiasis (n = 1), depression (n = 1), panic attack (n = 1), atrial septal defect (n = 1), chronic renal failure (n = 1). ^1^ HAE: hereditary angioedema, ^2^ FMF: familial Mediterranean fever, ^3^ AECT-3mth: angioedema control test-3 months, ^4^ pdC1-INH: plasma-derived C1 esterase inhibitor, ^5^ AEs: adverse events. NA: not applicable.

## Data Availability

The data presented in this study are available on request from the corresponding author due to legacy and ethical reasons.

## Abbreviations

The following abbreviations are used in this manuscript:

HAE	Hereditary angioedema
C1INH	C1 esterase inhibitor
HAE-C1INH	C1 esterase inhibitor deficiency and/or dysfunction
STP	Short-term prophylaxis
LTP	Long-term prophylaxis
pdC1-INH	plasma-derived C1 esterase inhibitors
FFP	Fresh frozen plasma
IQR	Interquartile range
AE	Angioedema
AECT-3 months	Angioedema control test-3 months
